# Association between childhood maltreatment and adult cortisol concentrations mediated through subjective health complaints

**DOI:** 10.3389/fepid.2023.1098822

**Published:** 2023-02-17

**Authors:** Johanna Klinger-König, Anke Hannemann, Nele Friedrich, Matthias Nauck, Henry Völzke, Hans J. Grabe

**Affiliations:** ^1^ Department of Psychiatry and Psychotherapy, University Medicine Greifswald, Greifswald, Germany; ^2^ Institute of Clinical Chemistry and Laboratory Medicine, University Medicine Greifswald, Greifswald, Germany; ^3^ German Centre for Cardiovascular Research (DZHK), Partner Site Greifswald, University Medicine Greifswald, Greifswald, Germany; ^4^ Institute for Community Medicine, University Medicine Greifswald, Greifswald, Germany; ^5^ German Center for Neurodegenerative Diseases (DZNE), Site Rostock/Greifswald, Greifswald, Germany

**Keywords:** trauma, abuse, neglect, depresion, obesity, cardiovascular diseas, mediatation

## Abstract

**Background:**

Lower cortisol concentrations in adulthood were repeatedly associated with more severe childhood maltreatment. Additionally, childhood maltreatment was reported to promote health risk behavior, such as smoking or alcohol consumption, and to increase the risk of mental and somatic diseases during adulthood, such as major depressive disorders or obesity. The present study investigated if health risk behavior and disease symptoms in adults mediate the associations between past childhood maltreatment and present basal serum cortisol concentrations.

**Methods:**

Data from two independent adult cohorts of the general population-based Study of Health in Pomerania (SHIP-TREND-0: *N* = 3,517; SHIP-START-2: *N* = 1,640) was used. Childhood maltreatment was assessed *via* the Childhood Trauma Questionnaire (CTQ). Cortisol concentrations were measured in single-point serum samples. Health risk behavior and mental and physical symptoms were used as mediators. Mediation analyses were calculated separately for both cohorts; results were integrated *via* meta-analyses.

**Results:**

In mediator-separated analyses, associations between childhood maltreatment and basal serum cortisol concentrations were partly mediated by depressive symptoms (BDI-II: *β*_indirect effect_ = -.011, *p*_FDR _= .017, 21.0% mediated) and subjective somatic health complaints (somatic complaints: *β*_indirect effect _= -.010, *p*_FDR _= .005, 19.4% mediated). In the second step, both mediators were simultaneously integrated into one mediation model. The model replicated the mediation effects of the subjective somatic health complaints (whole model: *β*_indirect effect _= -.014, *p* = .001, 27.6% mediated; BDI-II: *β*_indirect effect _= -.006, *p* = .163, 11.4% mediated, somatic complaints: *β*_indirect effect _= -.020, *p* = .020, 15.5% mediated).

**Conclusion:**

The results support the hypothesis that the long-lasting effects of childhood maltreatment on the stress response system are partly mediated through self-perceived disease symptoms. However, no mediation was found for health risk behavior or physically measured mediators. Mediation models with multiple simultaneous mediators pointed to a relevant overlap between the potential mediators. This overlap should be focused on in future studies.

## 1. Introduction

The World Health Organization (WHO) describes childhood maltreatment (CM) as the “abuse and neglect of children” (1). About 30% of the German adult population report CM (2, 3). Based on data from women enrolled in a US health maintenance organization, Walker et al. (4) reported a CM prevalence of 43%. These women reporting any CM also reported a worse subjective health status, more symptoms of somatic disorders or mental diseases as well as more health risk behaviors (4). As health risk factors, health risk behaviors subsume behavioral patterns or habits that increase the risk of somatic disorders or mental diseases.

*CM is a well-described risk factor for somatic diseases and mental disorders in adulthood* (5–7)*.* A meta-analysis by Hughes et al. (5) found associations between CM and health risk behaviors, mental disorders as well as somatic diseases. Besides an increase in health risk behaviors, such as smoking and problematic alcohol use, CM was repeatedly associated with various substance abuse disorders (5, 7, 8). Further mental disorders often associated with CM are mood disorders including major depressive disorder (MDD) and anxiety disorders including post-traumatic stress disorder (PTSD) (5–7, 9, 10). For most of these mental disorders, CM was found to lower the age of onset, worsen the course of symptoms, increase the treatment resistance and thus increase the risk of chronicity (6, 7, 10, 11).

Regarding somatic diseases, cardiovascular diseases as well as their risk factors were frequently described to be related to CM (12–15). Thus, on the one hand, CM was associated with myocardial infarction, stroke, and cardiovascular symptoms (13–15). On the other hand, associations with smoking, hypertension and obesity were observed (12–14, 16–18). Similar to mental symptoms, a dose-dependent increase in somatic symptoms after CM was reported (14, 15). Interestingly, the associations between CM and health risk factors for cardiovascular diseases such as physical symptoms including obesity or hypertension and health risk behaviors including smoking were found to be largely independent of each other (16, 18).

*In a regulatory manner, extreme or chronic stress may lead to an enhanced sensitivity of the hypothalamus-pituitary-adrenal (HPA) axis.* The HPA axis is the major endocrine stress response system regulating the secretion of glucocorticoids such as cortisol by the adrenal gland. In the absence of stress, cortisol is secreted in a circadian rhythm. Under stress conditions, the cortisol secretion is stimulated. An enhanced sensitivity of the HPA axis might prevent the body from chronically increased cortisol concentrations and prepare for faster stress responses in the light of future stress (19). Teicher and Samson (7) discussed the wide-ranging multifaceted impact of CM as a consequence of neurobiological changes. In rats, the neurotoxic effects of glucocorticoids on the forebrain were reported (20). In humans, glucocorticoids were observed to suppress neurogenesis in the hippocampus (7). Accordingly, chronic stress was associated with morphological and synaptic changes, particularly in the prefrontal cortex and subcortical regions such as the hippocampus, the amygdala and the hypothalamus (7, 21–24). These structures are also known as regulators of the HPA axis (7, 25). As an early stressor, CM was reported to have such prolonged effects on the HPA axis (26–28). Accordingly, CM was associated with lower basal blood and saliva cortisol concentrations (18, 26, 29–31).

*Altered basal cortisol concentrations were also reported for multiple CM-associated somatic diseases and mental disorders.* After burnout and exhaustion, alterations of the HPA axis were described that are similar to CM-associated changes (32, 33). Thus, lower basal blood cortisol concentrations were associated with more severe depressive symptoms, primarily in the absence of psychotic symptoms and in the comorbid presence of PTSD symptoms (19, 30, 34). Especially for cases with comorbid PTSD symptoms, it was discussed if these lower basal cortisol concentrations might rely on neurobiological changes due to the traumatic exposition (19). In MDD patients, however, higher blood cortisol concentrations predicted worse treatment outcomes (35). In contrast to CM and depressive symptoms, positive associations were reported between basal cortisol concentrations and health risk behaviors. Thus, smoking and high alcohol consumption were associated with higher basal cortisol concentrations (36–39). Moreover, Badrick et al. (36) did not observe any differences between current and ex-smokers suggesting long-term effects of smoking on the HPA axis.

Higher basal cortisol concentrations were also correlated with increased central obesity, particularly if obesity was reported as stress-induced (40–43). Accordingly, higher cortisol concentrations were associated with higher triglyceride concentrations and higher concentrations of high-density lipoproteins cholesterol (HDL-C), although results for the latter are more ambiguous (43). In a review, van Rossum (41) summarized that higher cortisol concentrations in obesity matched a higher risk for cardiovascular comorbidities. Indeed, as previously reported, higher systolic blood pressure was associated with both higher cortisol concentrations and obesity (40, 43). Whitworth et al. (43) even widened the hypothesis and reported an increased risk of cardiovascular factors facing elevated basal cortisol concentrations.

*Although, the effects of CM on health risk behavior, mental and somatic diseases as well as associations between these factors and cortisol concentrations are well described, investigating the triangle of CM, health factors and cortisol concentrations is just about to start.* Thus, lower basal cortisol concentrations after CM were mainly found to be independent of depressive symptoms (30, 31, 34). Prior studies demonstrated no additive effects of CM and depressive symptoms on basal cortisol concentrations and stress-dependent higher cortisol concentrations in obesity (30, 31, 34, 41, 42). Another study reported higher cortisol concentrations and higher systolic blood pressure to be associated with lower PTSD symptomatology in middle-aged and elderly patients (44).

Thus, on the one hand, basal cortisol concentrations are inversely associated with mental disorders but positively associated with somatic diseases on the other hand. These different effect directions for mental and somatic diseases need to be addressed. Accordingly, Meewisse et al. (45) reported lower cortisol concentrations in PTSD patients only if compared to non-exposed controls suggesting altered cortisol concentrations due to the exposure rather than the PTSD symptoms. As most diseases are researched in adulthood, mediation analyses with CM as exposure are advisable. As one of the first studies, Ju et al. (35) observed that the effects of CM on depressive symptoms in MDD patients were partly mediated through blood cortisol concentrations.

Analogous to Ju et al. (35), some studies used basal serum cortisol concentrations to longitudinally predict mental symptom severity or treatment outcomes (46, 47). Other studies, in contrast, provided evidence for the opposite effect direction: positive affect as well as mental symptoms longitudinally predicted basal cortisol changes (32, 33, 48, 49). In a recent review, Klimes-Dougan et al. (50) summarized the transgenerational effects of maternal depressive and bipolar disorders on the cortisol concentrations of the offspring. Due to circadian and ultra-circadian rhythms as well as interactions with other endogenous hormones, cortisol concentrations are likely more changeable than physical and mental symptoms. The present study integrated this assumption and the previous results while investigating the tringle of CM, basal blood cortisol concentrations and health issues *via* mediation analyses. Precisely, we assumed that the inverse association between CM and basal blood cortisol concentrations in adults of the general population is mediated by health risk behavior as well as physical and mental symptoms. Besides separate mediation analyses for each health issue, combined mediation analyses were calculated to account for the comorbidities of health risk behaviors, physical and mental symptoms. The analyses were based on two large independent samples of the general adult population of the northeast of Germany.

## 2. Materials and methods

### 2.1. Study population

Data from two independent cohorts of the Study of Health in Pomerania (SHIP) was used (51). SHIP comprises adult, general population-based samples which are drawn from local registries in the northeast of Germany. The assessments and examinations of the SHIP studies were approved by the institutional ethics review board of the University Greifswald. The data collection of the SHIP studies and all analyses of the present manuscript were performed according to the declaration of Helsinki including written informed consent of all participants.

The first SHIP cohort (SHIP-START-0: *N* = 4,308) was recruited between 1997 and 2001. Interview and medical examination data as well as laboratory measurements in blood samples of the 10-years-follow-up (SHIP-START-2: 2008–2012; *N* = 2,333) are included in the present analyses. All participants of SHIP-START-0 still alive in 2006 were re-invited to participate in the Study of Life Events and Gene-Environment-Interaction in Depression (SHIP-LEGEND: 2007–2010; *N* = 2,400). SHIP-LEGEND was used to assess life events and multiple psychiatric phenotypes in the SHIP-START sample with 1,944 participants assessed in both SHIP-START-2 and SHIP-LEGEND. Psychometric data of SHIP-LEGEND are used in the present analyses. Psychometric data (SHIP-LEGEND) was assessed about 19 months (SD = 14.3 months) before the interviews, medical examinations and blood samples (SHIP-START-2).

A second, independent cohort (SHIP-TREND-0; *N* = 4,420) was recruited between 2008 and 2012 in the same local area as SHIP-START. Participants of SHIP-START and SHIP-TREND are not overlapping. Interview, medical examination and questionnaire data of SHIP-TREND-0 were used in the present analyses as a second, independent sample.

### 2.2. Interview data

During a computer-assisted face-to-face interview, sociodemographic variables were assessed including sex and age. Cigarette smoking was assessed as either non-smoker, ex-smoker or current smoker; current and ex-smokers were summarized as ever-smokers for the present analyses. For ever-smokers, pack years were calculated according to Baumeister et al. (52). Alcohol consumption was quantified by the mean intake of ethanol in grams per day, averaged over the past 30 days (53). According to Baumeister et al. (53), risky alcohol consumption was defined as alcohol consumption of >20 g/d for women and >30 g/d for men. Participants were asked to bring their drug prescription sheet or medication containers and to report all medication used during the past week. Compounds were classified according to the Anatomical Therapeutic Chemical (ATC) classification (54).

### 2.3. Medical examination

Body height was measured to the nearest 1 cm. Body weight was measured in light close to the nearest 100 g. Waist circumference was measured to the nearest 1 mm with an inelastic tape midway between the lower rib margin and the iliac crest while the participant was standing plain on both feet. As a proxy for central body fat, the waist-height ratio was calculated (55). The body mass index (BMI) was used to define obesity with a BMI ≥30 classified as obese. Blood pressure (BP) was measured three times *via* a BP cuff on the upper arm. While the participant was sitting calmly, the first measurement was conducted after a five-minute rest; the second and third measurement was each conducted with a three-minute delay. The mean of the second and third BP measurements was used for statistical purposes. Hypertension was defined if the participant reported the intake of antihypertensive medication (ATC C02, ATC C03, ATC C07, ATC C08, ATC C09 or self-report) or if a systolic BP ≥140 mmHg or a diastolic BP ≥90 mmHg was measured.

### 2.4. Laboratory measurements

Single-point blood samples were taken from the cubital vein and stored at −80°C in the Integrated Research Biobank of the University Medicine Greifswald (56). The exact time of blood sampling was recorded. Participants of SHIP-TREND-0 but not participants of SHIP-START-2 were asked to fast before blood sampling. Fasting time was calculated in both cohorts by quantifying the time difference between the time of blood sampling and the time of the last reported caloric intake.

Processing of the serum samples to measure the cortisol concentrations is described elsewhere (57). Briefly, serum samples were prepared and frozen at −80°C directly after blood sampling. Samples were thawed and processed before the measurements. To quantify the serum cortisol concentrations, an immunoassay with low-cross reactivity was used on the AVIDA Centaur XP System (Siemens Healthcare Diagnostics, Eschborn, Germany). The coefficients of variation observed during the course of the measurements were low for both analyzed cohorts (30, 57).

White blood cell count (WBC) was measured either on the XT2000, XE 5000 or SE9000 analyzers from Sysmex (Sysmex Deutschland GmbH, Norderstedt, Germany) or on the Advia 2120i (Siemens Healthcare Diagnostics, Eschborn, Germany). Glycated hemoglobin (HbA1c) concentrations were quantified by high-performance liquid chromatography (Bio-Rad Diamat, Munich, Germany). Triglycerides were determined enzymatically (Dimension VISTA, Siemens Healthcare Diagnostics GmbH, Eschborn, Germany). High-density lipoprotein cholesterol (HDL-C) was measured enzymatically after the preparation with phosphotungstic acid/MgCl2.

All assays were performed by skilled personnel according to the manufacturer's instructions.

### 2.5. Psychometric data

CM was assessed *via* the Childhood Trauma Questionnaire (CTQ) (58). Based on 28 items, five subscales are assessed: emotional, physical and sexual abuse as well as emotional and physical neglect. Each item is answered on a five-point scale ranging from “never true” to “very often true”. A summary score operationalizes the severity of CM overall (range: 25–125) with higher values indicating more severe CM. Summary scores of the subscales can be categorized into “none”, “mild”, “moderate” or “severe”, often used to compare “none/mild” vs. “moderate/severe” CM (58, 59). Accordingly, dichotomous abuse and neglect scores were calculated as indicators of the absence or presence of CM for the present manuscript. The dichotomous scores thus extend the severity information given by the CTQ summary score.

To assess subjective health complaints, the Subjective Health Complaints (SHC) questionnaire was used (60). All symptoms are rated on a four-point scale. Based on the results of a factor analysis, 30 symptoms are assigned to eight clusters (60). According to Klinger-König et al. (61), the clusters anxiety/depression and exhaustion were subsumed as mental complaints and the remaining six clusters were subsumed as somatic complaints. For both categories the mental (range: 13–52) and the somatic SHC (range: 17–68) a summary score was calculated. For both scores, higher values indicate more severe health complaints.

Depressive symptoms were differently assessed in SHIP-TREND-0 and SHIP-START-2. Both questionnaires assessed the severity of depressive symptoms during the past two weeks. In SHIP-TREND-0, the depression module of the Patient Health Questionnaire was used (PHQ-9) (62). The PHQ-9 uses nine items that mimic the symptoms used as MDD diagnostic criteria in the Diagnostic and Statistical Manual of Mental Disorder - 4th Version (DSM-IV) (63). The experience of each symptom is rated on a four-point scale from “not at all” to “almost every day”. A summary score is calculated (range: 0–27) with higher values indicating more severe depressive symptoms. In SHIP-START-2, the Beck Depression Inventory-II (BDI-II) was used (64). The BDI-II uses 21 items to assess depressive symptoms. Each item is rated on a four-point scale formulated individually for each item. A summary score is calculated (range: 0–63) with higher values indicating more severe depressive symptoms.

### 2.6. Analytic sample

The selection of the analytic sample is presented in Supplementary Figure S1. Participants were excluded from analyses if data sets were incomplete for serum cortisol concentrations, the CTQ summary score, abuse and neglect, age, sex, fasting time, time of blood sampling, HbA1c and WBC (SHIP-TREND-0: *N* = 326; SHIP-START-2: *N* = 469). Further, participants were excluded if reporting the intake of corticosteroid medication (ATC H01, ATC H02, ATC R03; SHIP-TREND-0: *N* = 211; SHIP-START-2: *N* = 115) or sexual hormones or hormonal contraception (ATC G03, ATC G02B; SHIP-TREND-0: *N* = 366; SHIP-START-2: *N* = 109). Thus, the analytic sample comprised 3,517 participants for SHIP-TREND-0 and 1,640 participants for SHIP-START-2. Descriptive statistics of these analytic samples are provided in Table 1 and the results section. Descriptive statistics of covariates comparing the initial study samples with the analytic samples of both cohorts are presented in Supplementary Table S1.

**Table 1:** Descriptive statistics of the analytic samples of SHIP-TREND-0 and SHIP-START-2.

	SHIP-TREND-0		SHIP-START-2		
	*N*	M (SD)	*N*	M (SD)	*p*-value
COVARIATES					
Age (Years)	3,517	52.86 (14.76)	1,640	58.00 (13.05)	1.31e−27^a^
Sex (% Female)	3,517	46.83	1,640	48.90	0.169^b^
Fasting time (%)	3,517		1,640		1.55e−29^b^
<10:00		41.03		94.39	
10:00–12:00		16.38		1.22	
>12:00		42.59		4.39	
Time of Blood Sampling (h:min)	3,517	9:14 (1:04)	1,640	9:33 (1:01)	3.08e−31^a^
HbA1c (%)	3,517	5.38 (0.74)	1,640	5.51 (0.77)	1.20e−11^a^
WBC (Gpt/l)	3,517	6.02 (1.69)	1,640	6.02 (1.66)	0.831^a^
OUTCOME AND PREDICTORS					
Cortisol (nmol/l)	3,517	322.66 (116.08)	1,640	302.14 (110.83)	3.43e−10^a^
CTQ Summary Score	3,517	33.51 (9.66)	1,640	34.08 (9.82)	0.002^a^
CTQ Abuse (% Yes)	3,517	7.08	1,640	8.90	0.024^b^
CTQ Neglect (% Yes)	3,517	12.45	1,640	13.60	0.264^b^
MEDIATORS					
Systolic BP (mmHG)	3,507	128.26 (1.19)	1,635	133.00 (1.40)	<2.23e−31^a^
Hypertension (% Yes)	3,511	41.04	1,639	49.60	9.83e−09^b^
WHtR	3,505	0.54 (0.08)	1,637	0.55 (0.08)	8.62e−05^a^
Obesity (% Yes)	3,512	32.06	1,638	32.84	0.587^b^
Triglycerides (mmol/l)	3,501	1.63 (0.98)	1,631	1.86 (1.12)	7.63e−15^a^
HDL-C (mmol/l)	3,517	1.41 (0.37)	1,639	1.41 (0.37)	0.465^a^
Depressive Symptoms	3,472	3.87 (3.58)	1,640	6.18 (7.13)	6.75e−12^a^
Mental SHC	3,387	23.05 (7.50)	1,574	23.03 (7.53)	0.900^a^
Somatic SHC	3,375	27.77 (7.09)	1,572	27.94 (7.31)	0.572^a^
Alcohol Consumption (g/d)	3,483	8.95 (13.82)	1,530	10.55 (14.37)	1.73e−09^a^
Risky Alcohol Consumption (% Yes)	3,483	7.72	1,530	10.78	4.76e−04^b^
Pack Years	1,568	20.05 (16.67)	751	26.06 (64.05)	5.05e−04^a^
Ever-smoker (% Yes)	3,504	63.27	1,636	61.61	0.252^b^

HbA1c, glycated hemoglobin; WBC, white blood cell count; CTQ, Childhood Trauma Questionnaire; BP, blood pressure; WHtR, waist-height ratio; HDL-C, high-density lipoprotein cholesterol; SHC, Subjective Health Complaints.

^a^ Wilcoxon Rank Sum Test.

^b^ Fisher's Exact Test.

### 2.7. Statistical analyses

Statistical analyses were calculated with R 4.2.1 (65). For descriptive purposes, continuous variables are reported in mean (M) and standard deviation (SD); dichotomous variables are reported in percentage (%). All analyses were calculated separately for both cohorts in the first step and afterwards integrated *via* meta-analyses. Only meta-analytic results were interpreted.

In mediation analyses, serum cortisol concentrations were used as the outcome variable and the CTQ summary score, abuse and neglect were used as predictors. As potential mediators, the following variables were used: systolic BP, hypertension, WHtR, obesity, triglycerides, HDL-C, depressive symptoms, mental and somatic SHC, alcohol consumption, risky alcohol consumption, pack years and ever smoking. Association matrices of the mediators in SHIP-TREND-0 and SHIP-START-2 are presented in Supplementary Table S2. Associations between CM and serum cortisol concentrations, between CM and the potential mediator as well as between the potential mediators and serum cortisol concentrations are preconditioned to analyze statistical mediation effects. Hence, these associations were checked before calculating the mediation models. Highly skewed variables (skewness >|1|: CTQ summary score, systolic BP, triglycerides, depressive symptoms, alcohol consumption, pack years) were log-transformed (66). Log-transformation resulted in a distribution closer to normal. Specific sets of covariates were considered for each analysis as described below.

#### 2.7.1. Main effects

The main effects of CM and all mediators on serum cortisol concentrations were calculated using multiple linear regression models with robust standard errors. Separate analyses were conducted for each CM and mediator variable, respectively. To adjust for multiple testing, the false discovery rate (FDR) according to Benjamini and Hochberg (67) was used. All linear regression models were adjusted for age, sex, fasting time, time of blood sampling, HbA1c and WBC. Restricted cubic splines (RCS) with four knots (5th, 35th, 65th and 95th percentiles) were used to model non-linear associations between serum cortisol concentrations and age, fasting time as well as the time of blood sampling.

Associations between the CTQ summary score and all mediator variables were calculated *via* multiple linear regression models for continuous mediators and multiple logistic regression models for dichotomous mediators. Mediators were used as outcome variables. Robust standard errors were used. All analyses were adjusted for age (RCS) and sex. FDR (67) was used to adjust for multiple testing.

#### 2.7.2. Mediation models

Mediation models were calculated using the *mediate* function of the *psych* package (68). Standard errors were bootstrapped with 2,000 repetitions. Separate models for the three CM predictors were conducted. Firstly, separate mediation models were calculated for each suitable mediator. The analyses were adjusted for age (RCS), sex, fasting time (RCS), time of blood sampling (RCS), HbA1c and WBC. To adjust for multiple testing, the indirect effects were FDR-adjusted (67). Secondly, all mediators significant in the separate mediation models were integrated simultaneously into a full mediation model. Thus, the combined mediation effect of the mediators was calculated and the contribution of each mediator to this complete mediation effect was estimated. The covariates of the full mediation models equaled the separate mediation models.

#### 2.7.3. Sensitivity analyses

As the mental and somatic SHC scores are summarizing a large number of symptoms, sensitivity analyses on the impact of the 30 symptoms were conducted, Thus, the association between each item and CM and serum cortisol concentrations, respectively, was analyzed. Afterwards, symptoms significantly associated with both CM and serum cortisol concentrations entered separate mediation models. All sensitivity analyses were adjusted for age (RCS), sex, fasting time (RCS), time of blood sampling (RCS), HbA1c and WBC. *P*-values of both single linear regression models as well as indirect effects were FDR-adjusted to adjust for multiple testing (67).

## 3. Results

Descriptive statistics for SHIP-TREND-0 and SHIP-START-2 are provided in Table 1. The mean age of SHIP-TREND-0 participants was 53 years, while participants of SHIP-START-2 were 58 years old. The representation of men and women was similar in both samples (SHIP-TREND-0: 47% women; SHIP-START-2: 49% women). Participants of SHIP-TREND-0 had higher cortisol concentrations (SHIP-TREND-0: *M* = 322.7, SD = 116.1; SHIP-START-2: *M* = 302.1, SD = 110.8). Participants of SHIP-START-2 reported more severe CM. The mean differed by about half a point (SHIP-TREND-0: *M* = 33.5, SD = 9.7; SHIP-START-2: *M* = 34.1, SD = 9.8). Further, 2% more participants reported childhood abuse in SHIP-START-2 (SHIP-TREND-0: 7% abused; SHIP-START-2: 9% abused). Participants of both samples differed on most of the covariates and multiple mediators (Table 1).

### 3.1. Main effects

Associations between the CM variables and all potential mediators are provided in Table 2. Significant associations with the CTQ summary score were observed for WHtR, HDL-C, depressive symptoms, both SHC scores, alcohol consumption and pack years (*β* = −.037 – .260, all *p*_FDR _< .05). All associations were positive, except for HDL-C and alcohol consumption. For both abuse and neglect, associations were observed with depressive symptoms, both SHC scores and alcohol consumption (abuse: *β* = −.125 – .650, all *p*_FDR _< .05; neglect: *β* = −.153 – .439, all *p*_FDR _< .001). A positive association was observed between abuse and pack years (*β* = .195, *p*_FDR _= .012). For neglect, a positive association was found with WHtR (*β* = .094, *p*_FDR _= .045).

**Table 2:** Main effects of the CTQ summary score, abuse and neglect on potential mediators.

	CTQ Summary Score					CTQ Abuse					CTQ Neglect				
	T0	S2	Meta-analysis			T0	S2	Meta-analysis			T0	S2	Meta-analysis		
	*N*		*β*/OR	SE	*p*_FDR_	*N*		*β*/OR	SE	*p*_FDR_	*N*		*β*/OR	SE	*p*_FDR_
Systolic BP	3,507	1,635	0.034	0.028	0.217	3,507	1,635	−0.043	0.034	0.261	3,507	1,635	0.129	0.096	0.201
Hypertension	3,511	1,639	1.028	0.742	0.180	3,511	1,639	1.071	0.846	0.261	3,511	1,639	1.073	0.816	0.201
WHtR	3,505	1,637	0.035	0.013	0.022	3,505	1,637	0.073	0.052	0.256	3,505	1,637	0.094	0.040	0.045
Obesity (Yes)	3,512	1,638	1.056	0.740	0.180	3,512	1,638	1.178	0.833	0.256	3,512	1,638	1.272	0.805	0.165
Triglycerides	3,501	1,631	0.028	0.013	0.063	3,501	1,631	0.026	0.050	0.605	3,501	1,631	0.057	0.041	0.201
HDL-C	3,517	1,639	−0.030	0.013	0.038	3,517	1,639	−0.036	0.048	0.491	3,517	1,639	−0.079	0.038	0.081
Depressive Symptoms	3,472	1,640	0.260	0.013	2.51e−87	3,472	1,640	0.611	0.051	4.37e−32	3,472	1,640	0.437	0.042	1.50e−24
Mental SHC	3,387	1,574	0.254	0.016	5.45e−57	3,387	1,574	0.650	0.064	8.69e−24	3,387	1,574	0.439	0.048	3.82e−19
Somatic SHC	3,375	1,572	0.191	0.015	1.01e−35	3,375	1,572	0.432	0.058	5.51e−13	3,375	1,572	0.329	0.046	4.98e−12
Alcohol Consumption	3,483	1,530	−0.037	0.013	0.015	3,483	1,530	−0.125	0.051	0.037	3,483	1,530	−0.153	0.041	5.91e−04
Risky Alcohol Consumption (Yes)	3,483	1,530	1.103	0.761	0.180	3,483	1,530	1.153	0.941	0.261	3,483	1,530	1.125	0.879	0.201
Pack Years	1,568	751	0.080	0.018	1.99e−05	1,568	751	0.195	0.067	0.012	1,568	751	0.112	0.056	0.087
Ever-smoker (Yes)	3,504	1,636	1.204	0.740	0.150	3,504	1,636	1.690	0.843	0.097	3,504	1,636	1.498	0.811	0.105

*M*odels were adjusted for age (non-linear) and sex.

T0, SHIP-TREND-0; S2, SHIP-START-2; FDR, false discovery rate to adjust for multiple testing; CTQ, Childhood Trauma Questionnaire.

BP, blood pressure; WHtR, waist-height ratio; HDL-C, high-density lipoprotein cholesterol; SHC, Subjective Health Complaints.

All potential mediators were significantly associated with serum cortisol concentrations (*β* = −.180 – .273, all *p*_FDR_ < .05), except for systolic BP. For test statistics and FDR-adjusted *p*-values see Table 3. Positive associations were observed with hypertension, triglycerides, HDL-C and both alcohol consumption variables. Inverse associations were observed with all CM variables, WHtR, obesity, depressive symptoms, both SHC scores, pack years and ever smoking.

**Table 3:** Main effects on serum cortisol concentrations.

	T0	S2	Meta-analysis		
	*N*		*β*	SE	*p*_FDR_
**PREDICTORS**					
CTQ Summary Score	3,517	1,640	−0.052	0.012	7.25e−05
CTQ Abuse	3,517	1,640	−0.140	0.046	0.003
CTQ Neglect	3,517	1,640	−0.111	0.037	0.003
**MEDIATORS**					
Systolic BP	3,507	1,635	−0.002	0.004	0.509
Hypertension	3,511	1,639	0.103	0.031	0.001
WHtR	3,505	1,637	−0.087	0.016	8.95e−08
Obesity	3,512	1,638	−0.180	0.029	5.66e−09
Triglycerides	3,501	1,631	0.061	0.015	8.69e−05
HDL-C	3,517	1,639	0.108	0.015	4.02e−12
Depressive Symptoms	3,472	1,640	−0.051	0.013	2.56e−04
Mental SHC	3,387	1,574	−0.028	0.014	0.044
Somatic SHC	3,375	1,572	−0.059	0.014	8.69e−05
Alcohol Consumption	3,483	1,530	0.089	0.015	1.23e−08
Risky Alcohol Consumption	3,483	1,530	0.273	0.049	1.01e−07
Pack Years	1,568	751	−0.066	0.022	0.003
Ever-smoker	3,504	1,636	−0.108	0.028	2.06e−04

Models were adjusted for age (non-linear), sex, fasting time (non-linear), time of blood sampling (non-linear), glycated hemoglobin and white blood cell count.

T0, SHIP-TREND-0; S2, SHIP-START-2; FDR, false discovery rate to adjust for multiple testing.

CTQ, Childhood Trauma Questionnaire; BP, blood pressure; WHtR, waist-height ratio; HDL-C, high-density lipoprotein cholesterol; SHC, Subjective Health Complaints.

Accordingly, the following variables were included as potential mediators of the associations between CM and serum cortisol concentrations: (1) depressive symptoms, mental SHC, somatic SHC and alcohol consumption were tested for all CM variables, (2) WHtR for the CTQ summary score and neglect, (3) pack years for the CTQ summary score and abuse and (4) HDL-C for the CTQ summary score.

### 3.2. Mediation models

#### 3.2.1. Separate mediation models

**Figure 1:**
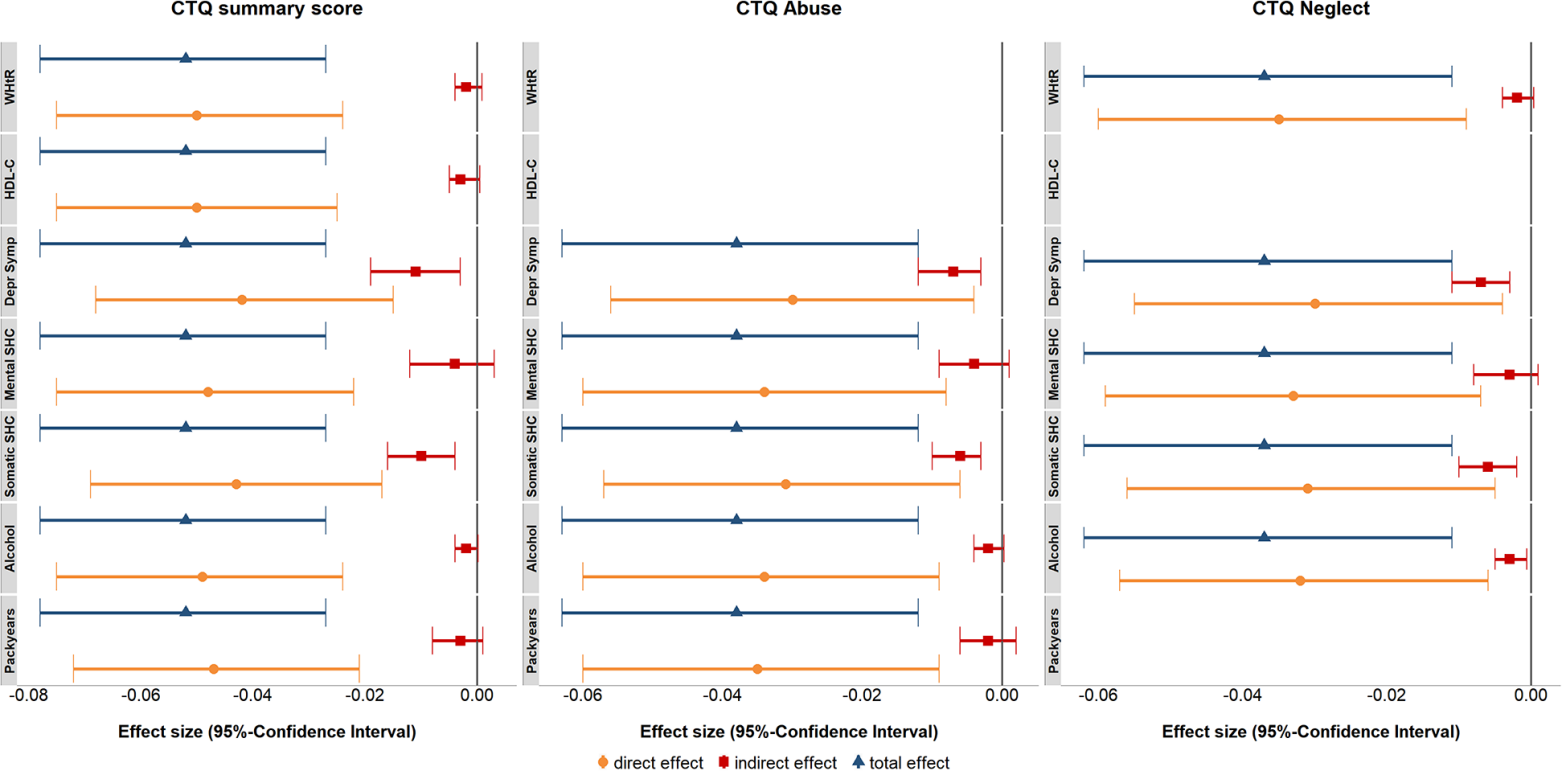
Forest plot of the effects of the separate mediation models for the CTQ summary score, abuse and neglect as predictors and serum cortisol concentrations as the outcome. The indirect effect describes the mediation effect through the mediator variable. The direct effect describes the effect of the predictor on serum cortisol concentrations adjusted for the mediator. The total effect describes the sum of the direct and indirect effects. All analyses were adjusted for age (non-linear), sex, fasting time (non-linear), time of blood sampling (non-linear), glycated hemoglobin and white blood cell count. CTQ, Childhood Trauma Questionnaire; WHtR, waist-height ratio; HDL-C, high-density lipoprotein cholesterol; Depr Symp. Depressive Symptoms; SHC, Subjective Health Complaints; Alcohol, Alcohol Consumption.

An overview of the results of the separate mediation models is presented in Figure 1 and Table 4.

**Table 4:** Separate mediation analyses for the impact of the CTQ summary score, abuse and neglect on serum cortisol concentrations.

	Total Effect			Direct Effect			Indirect Effect			
	*β*	SE	*p*-value	*β*	SE	*p*_FDR_	*β*	SE	*p*_FDR_	Proportion Mediated
CTQ Summary Score	−0.052	0.013	5.70e−05							
WHtR				−0.050	0.013	3.11e−04	−0.002	0.001	0.229	3.14
HDL-C				−0.050	0.013	3.11e−04	−0.003	0.002	0.171	4.79
Depressive Symptoms				−0.042	0.013	0.002	−0.011	0.004	0.019	21.04
Mental SHC				−0.048	0.013	4.58e−04	−0.004	0.004	0.281	7.84
Somatic SHC				−0.043	0.013	0.001	−0.010	0.003	0.007	19.24
Alcohol Consumption				−0.049	0.013	3.11e−04	−0.002	0.001	0.146	4.04
Pack Years				−0.047	0.013	4.58e−04	−0.003	0.002	0.225	5.99
CTQ Abuse	−0.038	0.013	0.004							
Depressive Symptoms				−0.030	0.013	0.023	−0.007	0.002	0.004	19.72
Mental SHC				−0.034	0.013	0.018	−0.004	0.003	0.146	10.71
Somatic SHC				−0.031	0.013	0.021	−0.006	0.002	0.004	17.22
Alcohol Consumption				−0.034	0.013	0.018	−0.002	0.001	0.146	5.00
Pack Years				−0.035	0.013	0.018	−0.002	0.002	0.322	5.24
CTQ Neglect	−0.037	0.013	0.005							
WHtR				−0.035	0.013	0.023	−0.002	0.001	0.118	5.54
Depressive Symptoms				−0.030	0.013	0.023	−0.007	0.002	0.005	19.07
Mental SHC				−0.033	0.013	0.023	−0.003	0.002	0.126	9.05
Somatic SHC				−0.031	0.013	0.023	−0.006	0.002	0.005	16.87
Alcohol Consumption				−0.032	0.013	0.023	−0.003	0.001	0.023	8.29

Models were adjusted for age (non-linear), sex, fasting time (non-linear), time of blood sampling (non-linear), glycated hemoglobin and white blood cell count.

FDR, false discovery rate to adjust for multiple testing; CTQ, Childhood Trauma Questionnaire; WHtR, waist-height ratio; HDL-C, high-density lipoprotein cholesterol; SHC, Subjective Health Complaints.

The impact of the CTQ summary score on serum cortisol concentrations was partly mediated by depressive symptoms (21.0% mediated, *p*_FDR _= .019) and the somatic SHC (19.2% mediated, *p*_FDR _= .007). The same structure of results was observed for the impact of abuse on serum cortisol concentrations. Again, depressive symptoms (19.7% mediated, *p*_FDR _= .004) and the somatic SHC (17.2% mediated, *p*_FDR _= .004) partly mediated the association. Similarly, the impact of neglect on serum cortisol concentrations was partly mediated by depressive symptoms (19.1% mediated, *p*_FDR _= .005) and the somatic SHC (16.9% mediated, *p*_FDR _= .005). The association between neglect and serum cortisol concentrations was partly mediated by alcohol consumption (8.3% mediated, *p*_FDR _= .023). Neither pack years nor alcohol consumption had a significant indirect effect on the CTQ summary score or abuse models. No mediation effects were observed for the mental SHC, WHtR or HDL-C.

#### 3.2.2 Full mediation models

**Figure 2:**
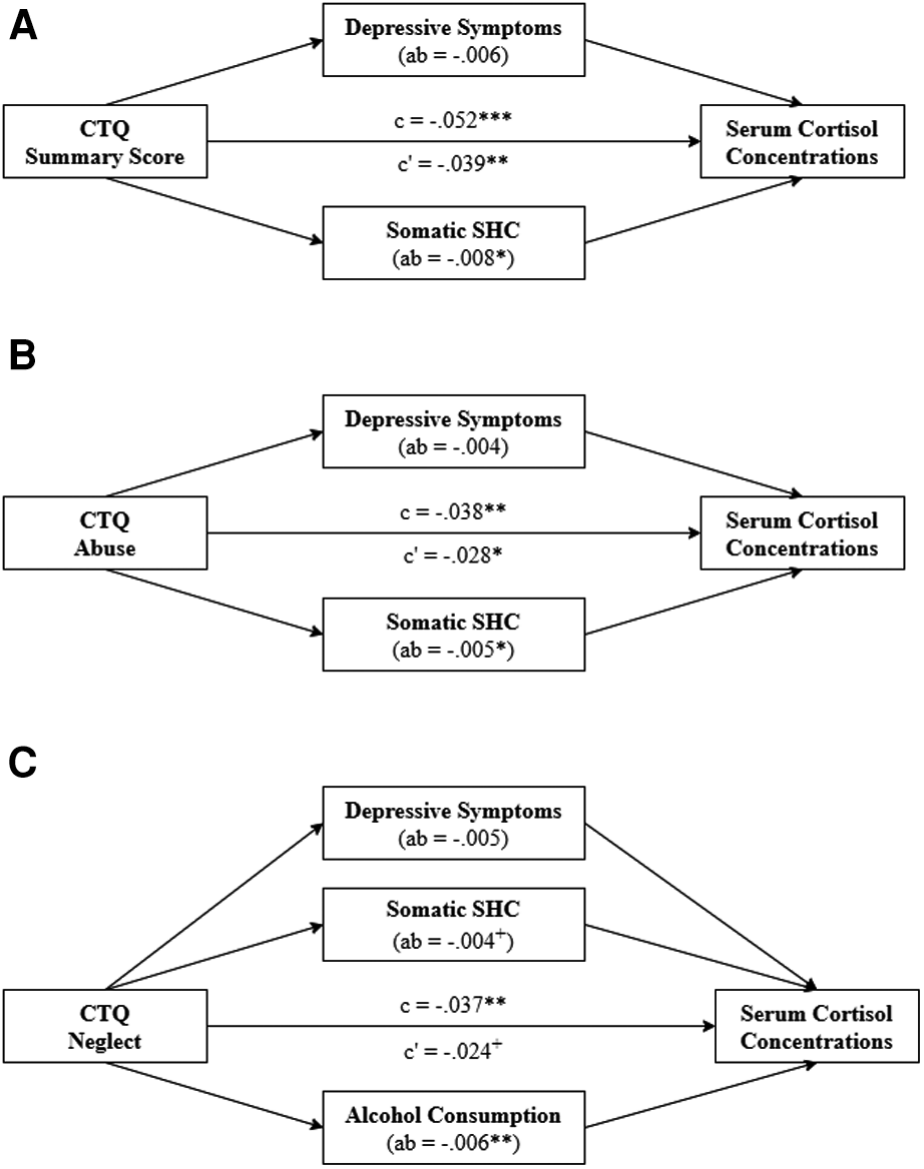
Full mediation models of the impact of the CTQ summary score (**A**), abuse (**B**) and neglect (**C**) on serum cortisol concentrations through depressive symptoms, the somatic SHC and alcohol consumption. The total effect (c) is the sum of the direct effect (c') and the indirect effects (ab) of both mediators. The direct effect describes the effect of the respective predictor on serum cortisol concentrations adjusted for all mediators. The indirect effects describes the mediation effects contributed by the respective mediator. All analyses were adjusted for age (non-linear), sex, fasting time (non-linear), time of blood sampling (non-linear), glycated hemoglobin and white blood cell count. CTQ, Childhood Trauma Questionnaire; SHC, Subjective Health Complaints; c, total effect; c′, direct effect; ab, indirect effect; ^+^*p* < .1; *p < .05; **p < .01; ***p < .001.

An overview of the results of the full mediation models is presented in Figure 2 and Table 5.

**Table 5:** Full mediation models for the impact of the CTQ summary score, abuse and neglect on serum cortisol concentrations.

	Total Effect			Direct Effect			Indirect Effect			
	*β*	SE	*p*-value	*β*	SE	*p*-value	*β*	SE	*p*-value	Proportion Mediated
CTQ Summary Score	−0.052	0.013	5.70e−05	−0.039	0.013	0.004	−0.014	0.004	0.002	27.68
Depressive Symptoms							−0.006	0.004	0.146	11.91
Somatic SHC							−0.008	0.003	0.018	15.46
CTQ Abuse	−0.038	0.013	0.004	−0.028	0.013	0.033	−0.009	0.003	7.77e−04	25.27
Depressive Symptoms							−0.004	0.003	0.107	11.00
Somatic SHC							−0.005	0.002	0.021	13.48
CTQ Neglect	−0.037	0.013	0.005	−0.024	0.013	0.067	−0.013	0.002	7.53e−08	35.47
Depressive Symptoms							−0.005	0.003	0.100	13.48
Somatic SHC							−0.004	0.002	0.088	12.02
Alcohol Consumption							−0.006	0.002	0.005	16.43

Models were adjusted for age (non-linear), sex, fasting time (non-linear), time of blood sampling (non-linear), glycated hemoglobin and white blood cell count.

CTQ, Childhood Trauma Questionnaire; SHC, Subjective Health Complaints.

For all three predictors, a mediation model including depressive symptoms and the somatic SHC as simultaneous mediators was calculated. Only for neglect, alcohol consumption was entered as the third mediator. A partial mediation of the impact of the predictor on serum cortisol concentrations through the combined mediators was observed in all models, i.e., for the CTQ summary score (27.7% mediated, *p*_ _= .002), abuse (25.3% mediated, *p*_ _= 7.77e-04) as well as neglect (35.5% mediated, *p*_ _= 7.53e-08). Focusing on the contribution of the single mediators, similar results were observed for the CTQ summary score and abuse: Whereas the partial mediation by the somatic SHC reached the significance level (CTQ summary score: 15.5% mediated, *p*_ _= .018; abuse: 13.5% mediated, *p*_ _= .021), the single mediation impact of depressive symptoms did not. In the neglect model, alcohol consumption had the largest contribution to the total mediation effect (16.4% mediated, *p*_ _= .005). The somatic SHC (12.0% mediated, *p*_ _= .088) was marginally significant (*p* < .1). The indirect effect of depressive symptoms did not reach the significance level.

### 3.3. Sensitivity analyses

**Figure 3:**
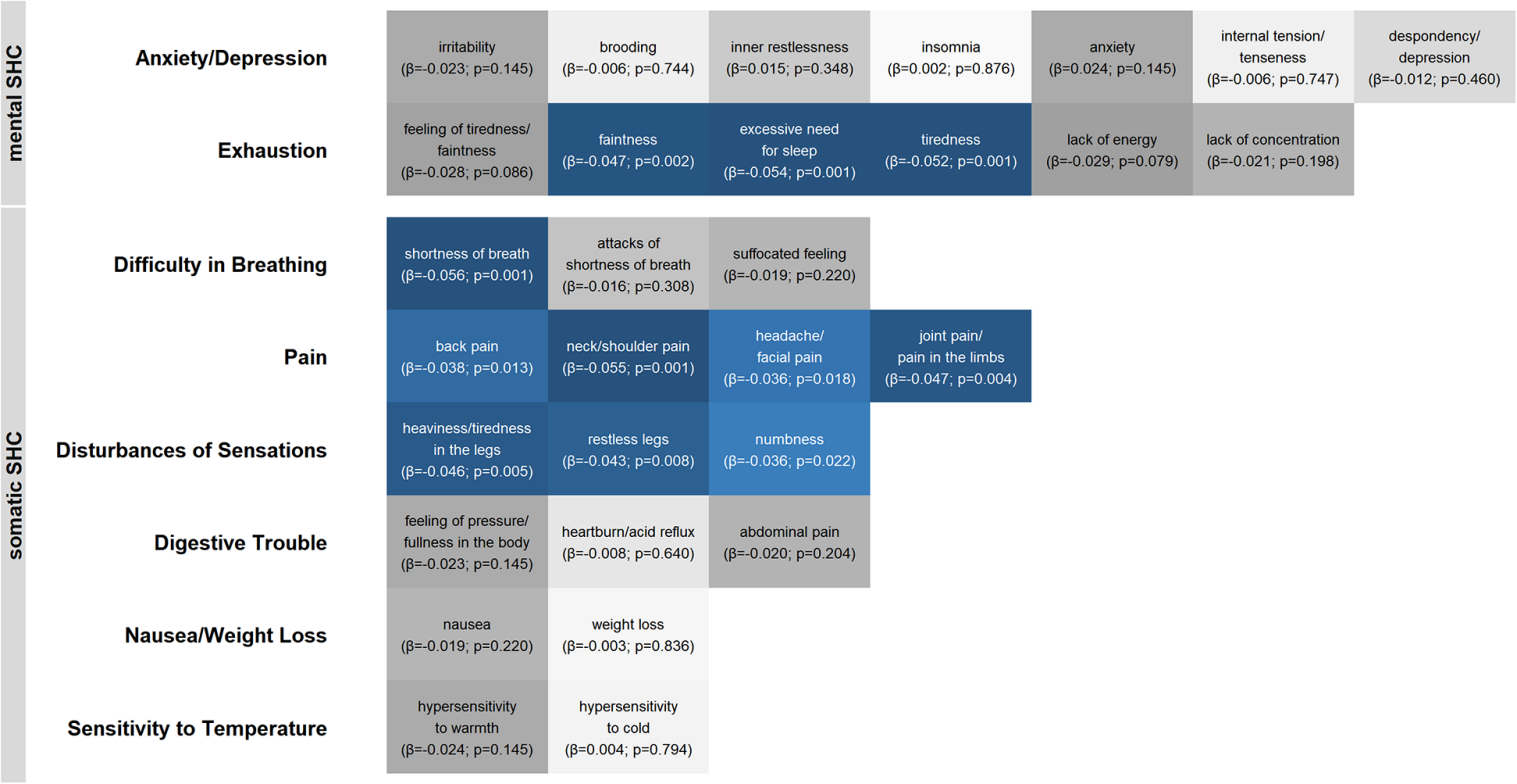
Heat map for the associations between serum cortisol concentrations and the symptoms of the subjective health complaints questionnaire. The symptoms are assigned to eight symptom clusters named at the y axis. Anxiety/depression and exhaustion were summarized as mental complaints; the remaining six clusters were summarized as somatic complaints. Blue tiles represent significant associations; gray tiles represent insignificant associations. Darker colors represent smaller p-values. All presented p-values are FDR-adjusted. All analyses were adjusted for age (non-linear), sex, fasting time (non-linear), time of blood sampling (non-linear), glycated hemoglobin and white blood cell count. SHC, Subjective Health Complaints.

All 30 symptoms of the SHC were associated with the CTQ summary score (*β* = .065 – .219, all *p*_FDR _< .001) and abuse (*β* = .130 – .590, all *p*_FDR _< .05). For neglect, a significant association was observed for all symptoms (*β* = .092 – .419, all *p*_FDR _< .05), except for suffocated feeling (*β* = .088, *p*_FDR _= .066). Nevertheless, only eleven symptoms were associated with serum cortisol concentrations. These items covered four symptom clusters: exhaustion, difficulty breathing, pain and disturbances of sensations. The results of the associations between the SHC symptoms and serum cortisol concentrations are presented in Figure 3. As suffocated feeling was not associated with serum cortisol concentrations, all eleven symptoms significantly associated with serum cortisol concentrations were tested as potential mediators of the associations with the CTQ summary score, abuse and neglect, respectively.

The results of the separate mediation analyses are presented in Supplementary Table S3. The impact of the CTQ summary score on serum cortisol concentrations was partly mediated by all symptoms (5.86–13.72% mediated, all *p*_FDR _< .05). The impact of abuse on serum cortisol concentrations was partly mediated by the three symptoms of exhaustion (13.49–14.69% mediated, all *p*_FDR _= .012) and shortness of breath (8.65% mediated, *p*_FDR _= .028). However, no indirect effect was observed for headache/facial pain, joint pain/pain in the limbs and numbness. A similar structure of the results was found for neglect: The association between neglect and serum cortisol concentrations was partly mediated by the three symptoms of exhaustion (8.65%–11.01% mediated, all *p*_FDR _= .038) and shortness of breath (6.53% mediated, *p*_FDR _= .049). No indirect effect was observed for neck/shoulder pain, headache/facial pain, joint pain/pain in the limbs, heaviness/tiredness in the legs and numbness.

## 4. Discussion

The association between CM and lowered basal cortisol concentrations is well described and was replicated in our data (18, 26, 29–31). In addition, the present study investigated if this association was mediated by health risk behaviors, mental or physical symptoms. The variety of potential mediators tested was based on previous research. However, mandatory associations with both CM as the predictor and serum cortisol concentrations as the outcome were only observed for a few disease symptoms. Among those, depressive symptoms and somatic SHC were the most robust mediators.

The triangle of CM, depressive symptoms and the HPA axis is well described (30, 31, 34, 35). Analog to previous results, our data demonstrated that CM and more severe depressive symptoms were both associated with lower basal cortisol concentrations, accomplished by a strong positive association between CM and depressive symptoms themselves. According to the sensitization theory of Heim et al. (19), a biological adaptation to CM might induce a long-term reduction of basal cortisol concentrations to prevent chronically enhanced cortisol concentrations. Simultaneously, the HPA axis is sensitized to new stressors resulting in a potentiated stress reaction (19). Similar alterations of the HPA axis were observed after burnout and exhaustion (32, 33). In line, CM increases the risk of mental symptoms and mental disorders including MDD (6, 9, 10). In MDD patients, more severe CM and higher basal cortisol concentrations were associated with worse courses of symptoms and worse treatment outcomes (6, 11, 35).

However, using adjusted linear regression models, the association between CM and cortisol concentrations was found to be independent of depressive symptoms (30, 31, 34). On the other hand, Ju et al. (35) observed a mediation of the association between CM and MDD severity by cortisol concentrations and dysfunctional attitudes in MDD patients. The present analyses were based on a general population sample with low CM severity and low severity of depressive symptoms. Our results supported both postulated relations: We observed a partial mediation of the association between CM and cortisol concentrations by depressive symptoms. Hence, lowered basal serum cortisol concentrations after CM were partly explained by more severe depressive symptoms. At the same time, a weaker, though depression-independent association between CM and cortisol concentrations was observed.

The mediation effect of depressive symptoms was lowered by simultaneously including somatic SHC as a second mediator. Interestingly, depressive symptoms did not diminish the indirect effect of somatic SHC. The somatic SHC comprised nausea and weight loss. In earlier studies, nausea and weight loss were associated with a worse subjective mental health status (60). Further, changes in appetite and weight are rated as depressive symptoms (62, 64). A moderate association between more somatic SHC and more severe depressive symptoms was previously also reported from SHIP (61). Accordingly, in parallel to the partial mediation effect by depressive symptoms, we observed a partial mediation of the association between CM and serum cortisol concentrations by somatic SHC.

Mental SHC, on the other hand, had no mediating effect in our analyses, although moderate associations between mental SHC, somatic SHC and depressive symptoms were reported in previous studies (60, 61). Our main effect analyses revealed a much smaller association between mental SHC and basal serum cortisol concentrations compared to associations between basal serum cortisol concentrations and depressive symptoms or somatic SHC. From a statistical point of view, this lowers the probability of a large mediation effect (69). From a theoretical point of view, mental SHC in our analyses combined depressive symptoms, anxiety and exhaustion. Sensitivity analyses revealed that some of the symptoms subsumed as exhaustion were associated with serum cortisol concentrations and showed a robust partial mediation of the association between CM and serum cortisol concentrations. In contrast, no association with serum cortisol concentrations were observed for the symptoms subsumed as anxiety/depression. Whereas more depressive symptoms and exhaustion were associated with lower basal cortisol concentrations (30–32, 34), the evidence of altered basal cortisol concentrations associated with anxiety is more ambiguous.

Regarding anxiety, Keefe et al. (70) reported associations between lower basal saliva cortisol concentrations with more severe generalized anxiety symptoms. In contrast, Mantella et al. (71) reported higher basal saliva cortisol concentrations together with more severe generalized anxiety symptoms in elderly patients. Meta-analysis investigating the associations between cortisol concentrations and PTSD did not reveal any overall effect (44, 45). However, subgroup analyses demonstrated an age shift at 30 years: PTSD was associated with higher cortisol concentrations in adolescents and young adults but lower cortisol concentrations in middle-aged and elderly adults (44). Additionally, analyses by Meewisse et al. (45) demonstrated that effects on cortisol concentrations might be caused by the exposure rather than the effect of PTSD.

In sum, mental symptoms as well as SHC rely on the subjective evaluation of perceived symptoms and well-being, while biomarkers and somatic disorders used as somatic mediators in our study, like systolic BP, obesity or HDL-C, are more objective. Although previously reported associations between these physical symptoms and serum cortisol concentrations were replicated (40, 41, 43), only a few associations with CM were observed. According to a review and meta-analysis by Norman et al. (72), associations between CM and somatic diseases are smaller than associations between CM and mental disorders. Additionally, Fukuda and Morimoto (37) summarized in a review that not obesity itself but weight change was associated with altered basal cortisol concentrations. Similar to weight change, the length of depressive episodes is rather short, mostly lasting for weeks or a few months (73, 74).

Importantly, mental and physical symptoms are interacting with each other. Thus, mentally diseased patients are more likely obese and have a higher risk of cardiovascular diseases and even mortality (75–78). Both mental and physical symptoms were previously associated with health risk behaviors such as smoking and alcohol consumption (78–81). Smoking and alcohol consumption were also associated with CM and basal cortisol concentrations before (5, 7, 8, 36, 37, 39). Accordingly, health risk behaviors were integrated as potential mediators in the present analyses with the expectancy of great effects.

However, alcohol consumption was the only health risk behavior assessed that partially mediated the effect between childhood neglect and basal serum cortisol concentrations. Interestingly, the indirect effect of alcohol consumption was low in single mediation analyses but increased when integrated into the model simultaneously with depressive symptoms and somatic SHC. This might hint at an upstream mediation effect with more severe CM encouraging health risk behaviors, which in turn promote depressive symptoms and subjective health complaints. Nevertheless, future research is needed to test this hypothesis. For smoking, pack years rather than smoking status was associated with CM. Yet, no mediation effect was observed for either variable.

### Strengths and limitations

Conclusively, our analyses were based on a general population sample with a rather low CM and symptom severity. We used two large and independent cohorts and integrated the results *via* meta-analysis which makes the overall results statistically more robust. The meta-analytic approach also enabled us to overcome differences between the samples, e.g., different fasting times. Nevertheless, the tested associations might be more prone in patient samples. Additionally, future studies should aim at longitudinal instead of cross-sectional data to support the mediation direction not only by theory but also *via* data structure.

Our analyses used basal serum cortisol concentrations as the outcome. Thus, the results are focused rather on the basic functioning of the HPA axis than on stress reactivity. Moreover, we used single-point cortisol measurements. Repeated individual cortisol measurements would allow to assess diurnal slopes or calculate the area under the curve and thus enable the use of a more robust outcomes. Nevertheless, the measured serum cortisol concentrations were validated in a multi-omics study including cortisol-associated transcriptome concentrations (82). Here, the exact time of blood sampling was included as a covariate and modeled non-linear to adjust for the circadian rhythm of cortisol secretion. Further, we excluded participants with drug use potentially inferring with the cortisol secretion.

The mediators included in the present analyses interact with each other. Thus, a more complex mediation model might be needed to display the pathways. We aimed to fit a wide range of behaviors, disease symptoms and diseases potentially mediating the association between CM and basal cortisol concentrations. We focused on previously well-described variables and our analyses provide a first overview integrating these potential mediators in one study. Although the selection of potential mediators was theory driven, the description of suitable mediators was based on *p*-values and rather exploratory. This approach enabled to extend previous research by integrating and comparing a wide set of potential mediators as well as to narrow this set down. However, focusing on a narrower set would ease the integration of multiple mediators in more complex models.

Mental symptoms were represented by mental SHC and depressive symptoms. Anxiety symptoms or anxiety disorders were not included in the present study as these were insufficiently assessed. Besides, depressive symptoms and SHC were assessed by self-report questionnaires. Interviews and, validated diagnoses particularly for mental disorders, could improve the data validity. Further, similar mediation effects should be addressed with other mental symptoms and disorders in future studies. As Ju et al. (35) reported a mediation by dysfunctional attitudes, integrating personality traits as a mediator might also be promising.

Finally, although the used questionnaire limits CM to childhood and adolescents, no exact time of CM was assessed. Thus, a potential difference between early or late CM could not be evaluated. However, including CM-related timing information in future studies and differentiating between types of CM could extend our presented results.

### Conclusion

The present study investigated a wide range of potential mediators for the association between CM and basal cortisol concentrations. Our analyses assessed several mediators described in multiple studies before, but followed a more integrative approach. In our two large general population-based samples, associations between the potential mediators and CM were largely absent. Thus, CM seem to add up to the effects of both physical and mental symptoms and should be addressed in both psychiatric and somatic medicine. Yet, mediation effects of depressive symptoms and somatic SHC were robust. Hence, especially in psychiatric medicine, the physiological impact of mental diseases should be addressed in the light of childhood maltreatment. Finally, our results are a first step towards more complex models representing the nuanced structure between the tested mediators.

## Data Availability

The data analyzed in this study is subject to the following licenses/restrictions: Data are applicable *via* the Transfer Unit for Data and Biomaterials of the University Medicine Greifswald. The authors do not have the permission to share the data. More detailed information on data application is provided at https://www.fvcm.med.uni-greifswald.de/cm_antrag/index.php?s=startseite&amp;lang=eng.

## References

[1] World Health Organization. Global status report on preventing violence against children. Geneva: World Health Organization (2020).

[2] Witt A Brown RC Plener PL Brähler E Fegert JM. Child maltreatment in Germany: prevalence rates in the general population. Child Adolesc Psychiatry Ment Health. (2017) 11:47. 10.1186/s13034-017-0185-0 28974983 PMC5621113

[3] Klinger-König J Streit F Erhardt A Kleineidam L Schmiedek F Schmidt B The assessment of childhood maltreatment and its associations with affective symptoms in adulthood: results of the German National Cohort (NAKO). World J Biol Psychiatry. (2022):1–12. 10.1080/15622975.2021.2011406 35302904

[4] Walker EA Gelfand A Katon WJ Koss MP von Korff M Bernstein D Adult health status of women with histories of childhood abuse and neglect. Am J Med. (1999) 107:332–9. 10.1016/S0002-9343(99)00235-1 10527034

[5] Hughes K Bellis MA Hardcastle KA Sethi D Butchart A Mikton C The effect of multiple adverse childhood experiences on health: a systematic review and meta-analysis. Lancet Public Health. (2017) 2:e356–66. 10.1016/S2468-2667(17)30118-4 29253477

[6] Nelson J Klumparendt A Doebler P Ehring T. Childhood maltreatment and characteristics of adult depression: meta-analysis. Br J Psychiatry. (2017) 210:96–104. 10.1192/bjp.bp.115.180752 27908895

[7] Teicher MH Samson JA. Childhood maltreatment and psychopathology: a case for ecophenotypic variants as clinically and neurobiologically distinct subtypes. Am J Psychiatry. (2013) 170:1114–33. 10.1176/appi.ajp.2013.12070957 23982148 PMC3928064

[8] Tonmyr L Thornton T Draca J Wekerle C. A review of childhood maltreatment and adolescent substance use relationship. CPSR. (2010) 6:223–34. 10.2174/157340010791792581

[9] Gardner MJ Thomas HJ Erskine HE. The association between five forms of child maltreatment and depressive and anxiety disorders: a systematic review and meta-analysis. Child Abuse Negl. (2019) 96:104082. 10.1016/j.chiabu.2019.104082 31374447

[10] Struck N Krug A Yuksel D Stein F Schmitt S Meller T Childhood maltreatment and adult mental disorders—the prevalence of different types of maltreatment and associations with age of onset and severity of symptoms. Psychiatry Res. (2020) 293:113398. 10.1016/j.psychres.2020.113398 32920524

[11] Nanni V Uher R Danese A. Childhood maltreatment predicts unfavorable course of illness and treatment outcome in depression: a meta-analysis. Am J Psychiatry. (2012) 169:141–51. 10.1176/appi.ajp.2011.11020335 22420036

[12] Chandan JS Okoth K Gokhale KM Bandyopadhyay S Taylor J Nirantharakumar K. Increased cardiometabolic and mortality risk following childhood maltreatment in the United Kingdom. J Am Heart Assoc. (2020) 9:e015855. 10.1161/JAHA.119.015855 32410481 PMC7660837

[13] Basu A McLaughlin KA Misra S Koenen KC. Childhood maltreatment and health impact: the examples of cardiovascular disease and type 2 diabetes mellitus in adults. Clin Psychol (New York). (2017) 24:125–39. 10.1111/cpsp.12191 28867878 PMC5578408

[14] Clemens V Huber-Lang M Plener PL Brähler E Brown RC Fegert JM. Association of child maltreatment subtypes and long-term physical health in a German representative sample. Eur J Psychotraumatol. (2018) 9:1510278. 10.1080/20008198.2018.1510278 30220980 PMC6136347

[15] Chou P-H Koenen KC. Associations between childhood maltreatment and risk of myocardial infarction in adulthood: results from the national epidemiologic survey on alcohol and related conditions. J Psychiatr Res. (2019) 116:172–7. 10.1016/j.jpsychires.2018.12.001 30553535

[16] Danese A Tan MT. Childhood maltreatment and obesity: systematic review and meta-analysis. Mol Psychiatry. (2014) 19:544–54. 10.1038/mp.2013.54 23689533

[17] Taha F Galea S Hien D Goodwin RD. Childhood maltreatment and the persistence of smoking: a longitudinal study among adults in the US. Child Abuse Negl. (2014) 38:1995–2006. 10.1016/j.chiabu.2014.10.022 25466425 PMC4448710

[18] Deighton S Neville A Pusch D Dobson K. Biomarkers of adverse childhood experiences: a scoping review. Psychiatry Res. (2018) 269:719–32. 10.1016/j.psychres.2018.08.097 30273897

[19] Heim CM Newport DJ Mletzko T Miller AH Nemeroff CB. The link between childhood trauma and depression: insights from HPA axis studies in humans. Psychoneuroendocrinology. (2008) 33:693–710. 10.1016/j.psyneuen.2008.03.008 18602762

[20] Alonso G. Prolonged corticosterone treatment of adult rats inhibits the proliferation of oligodendrocyte progenitors present throughout white and gray matter regions of the brain. Glia. (2000) 31:219–31. 10.1002/1098-1136(200009)31:3<219:AID-GLIA30>3.0.CO;2-R 10941148

[21] Pittenger C Duman RS. Stress, depression, and neuroplasticity: a convergence of mechanisms. Neuropsychopharmacology. (2008) 33:88–109. 10.1038/sj.npp.1301574 17851537

[22] Rajkowska G. Postmortem studies in mood disorders indicate altered numbers of neurons and glial cells. Biol Psychiatry. (2000) 48:766–77. 10.1016/S0006-3223(00)00950-1 11063973

[23] Groeneweg FL Karst H de Kloet ER Joëls M. Rapid non-genomic effects of corticosteroids and their role in the central stress response. J Endocrinol. (2011) 2:153–67. 10.1530/JOE-10-0472 21357682

[24] Dannlowski U Stuhrmann A Beutelmann V Zwanzger P Lenzen T Grotegerd D Limbic scars: long-term consequences of childhood maltreatment revealed by functional and structural magnetic resonance imaging. Biol Psychiatry. (2012) 71:286–93. 10.1016/j.biopsych.2011.10.021 22112927

[25] Nieh EH Kim S-Y Namburi P Tye KM. Optogenetic dissection of neural circuits underlying emotional valence and motivated behaviors. Brain Res. (2013) 1511:73–92. 10.1016/j.brainres.2012.11.001 23142759 PMC4099056

[26] Fogelman N Canli T. Early life stress and cortisol: a meta-analysis. Horm Behav. (2018) 98:63–76. 10.1016/j.yhbeh.2017.12.014 29289660

[27] Bublitz MH Parade S Stroud LR. The effects of childhood sexual abuse on cortisol trajectories in pregnancy are moderated by current family functioning. Biol Psychol. (2014) 103:152–7. 10.1016/j.biopsycho.2014.08.014 25220484 PMC4258150

[28] Yehuda R Halligan SL Grossman R. Childhood trauma and risk for PTSD: relationship to intergenerational effects of trauma, parental PTSD, and cortisol excretion. Dev Psychopathol. (2001) 13:733–53. 10.1017/S0954579401003170 11523857

[29] Hakamata Y Mizukami S Izawa S Moriguchi Y Hori H Matsumoto N Childhood trauma affects autobiographical memory deficits through basal cortisol and prefrontal-extrastriate functional connectivity. Psychoneuroendocrinology. (2021) 127:105172. 10.1016/j.psyneuen.2021.105172 33831650

[30] Klinger-König J Frenzel S Hannemann A Wittfeld K Bülow R Friedrich N Sex differences in the association between basal serum cortisol concentrations and cortical thickness. Neurobiol Stress. (2021) 15:100416. 10.1016/j.ynstr.2021.100416 34786441 PMC8578044

[31] O’Connor DB Green JA Ferguson E O’Carroll RE O’Connor RC. Effects of childhood trauma on cortisol levels in suicide attempters and ideators. Psychoneuroendocrinology. (2018) 88:9–16. 10.1016/j.psyneuen.2017.11.004 29144990

[32] Kudielka BM Bellingrath S Hellhammer DH. Cortisol in burnout and vital exhaustion: an overview. G Ital Med Lav Ergon. (2006) 28:34–42. PMID-ID: 19031555 19031555

[33] Kudielka BM von Känel R Preckel D Zgraggen L Mischler K Fischer JE. Exhaustion is associated with reduced habituation of free cortisol responses to repeated acute psychosocial stress. Biol Psychol. (2006) 72:147–53. 10.1016/j.biopsycho.2005.09.001 16236419

[34] Heim C Newport DJ Bonsall R Miller AH Nemeroff CB. Altered pituitary-adrenal axis responses to provocative challenge tests in adult survivors of childhood abuse. Am J Psychiatry. (2001) 158:575–81. 10.1176/appi.ajp.158.4.575 11282691

[35] Ju Y Wang M Lu X Sun J Dong Q Zhang L The effects of childhood trauma on the onset, severity and improvement of depression: the role of dysfunctional attitudes and cortisol levels. J Affect Disord. (2020) 276:402–10. 10.1016/j.jad.2020.07.023 32871670

[36] Badrick E Kirschbaum C Kumari M. The relationship between smoking status and cortisol secretion. J Clin Endocrinol Metab. (2007) 92:819–24. 10.1210/jc.2006-2155 17179195

[37] Fukuda S Morimoto K. Lifestyle, stress and cortisol response: review II lifestyle. Environ Health Prev Med. (2001) 6:15–21. 10.1007/BF02897304 21432232 PMC2723649

[38] Steptoe A Ussher M. Smoking, cortisol and nicotine. Int J Psychophysiol. (2006) 59:228–35. 10.1016/j.ijpsycho.2005.10.011 16337291

[39] Thayer JF Hall M Sollers JJ Fischer JE. Alcohol use, urinary cortisol, and heart rate variability in apparently healthy men: evidence for impaired inhibitory control of the HPA axis in heavy drinkers. Int J Psychophysiol. (2006) 59:244–50. 10.1016/j.ijpsycho.2005.10.013 16325293

[40] Abraham SB Rubino D Sinaii N Ramsey S Nieman LK. Cortisol, obesity, and the metabolic syndrome: a cross-sectional study of obese subjects and review of the literature. Obesity. (2013) 21:E105–17. 10.1002/oby.20083 23505190 PMC3602916

[41] van Rossum EF. Obesity and cortisol: new perspectives on an old theme. Obesity. (2017) 25:500–1. 10.1002/oby.21774 28229549

[42] Vicennati V Pasqui F Cavazza C Pagotto U Pasquali R. Stress-related development of obesity and cortisol in women. Obesity (Silver Spring). (2009) 17:1678–83. 10.1038/oby.2009.76 19300426

[43] Whitworth JA Williamson PM Mangos G Kelly JJ. Cardiovascular consequences of cortisol excess. Vasc Health Risk Manag. (2005) 1:291–9. 10.2147/vhrm.2005.1.4.291 17315601 PMC1993964

[44] Morris MC Hellman N Abelson JL Rao U. Cortisol, heart rate, and blood pressure as early markers of PTSD risk: a systematic review and meta-analysis. Clin Psychol Rev. (2016) 49:79–91. 10.1016/j.cpr.2016.09.001 27623149 PMC5079809

[45] Meewisse M-L Reitsma JB de Vries G-J Gersons BP Olff M. Cortisol and post-traumatic stress disorder in adults: systematic review and meta-analysis. BJP. (2007) 191:387–92. 10.1192/bjp.bp.106.024877 17978317

[46] Shirtcliff EA Essex MJ. Concurrent and longitudinal associations of basal and diurnal cortisol with mental health symptoms in early adolescence. Dev Psychobiol. (2008) 50:690–703. 10.1002/dev.20336 18726897 PMC2660275

[47] Fischer S Strawbridge R Vives AH Cleare AJ. Cortisol as a predictor of psychological therapy response in depressive disorders: systematic review and meta-analysis. BJP. (2017) 210:105–9. 10.1192/bjp.bp.115.180653 27908897

[48] Hoyt LT Craske MG Mineka S Adam EK. Positive and negative affect and arousal: cross-sectional and longitudinal associations with adolescent cortisol diurnal rhythms. Psychosom Med. (2015) 77:392–401. 10.1097/PSY.0000000000000178 25905661 PMC4431930

[49] Lupien S Lecours AR Schwartz G Sharma S Hauger RL Meaney MJ Longitudinal study of basal cortisol levels in healthy elderly subjects: evidence for subgroups. Neurobiol Aging. (1996) 17:95–105. 10.1016/0197-4580(95)02005-5 8786810

[50] Klimes-Dougan B Papke V Carosella KA Wiglesworth A Mirza SA Espensen-Sturges TD Basal and reactive cortisol: a systematic literature review of offspring of parents with depressive and bipolar disorders. Neurosci Biobehav Rev. (2022) 135:104528. 10.1016/j.neubiorev.2022.104528 35031342

[51] Völzke H Schössow J Schmidt CO Jürgens C Richter A Werner A Cohort profile update: the study of health in pomerania (SHIP). Int J Epidemiol. (2022)51:e372–e383. 10.1093/ije/dyac034 35348705

[52] Baumeister SE Schumann A Meyer C John U Völzke H Alte D. Effects of smoking cessation on health care use: is elevated risk of hospitalization among former smokers attributable to smoking-related morbidity? Drug Alcohol Depend. (2007) 88:197–203. 10.1016/j.drugalcdep.2006.10.015 17118577

[53] Baumeister SE Alte D Meyer C John U. Riskanter Alkoholkonsum und alkoholbezogene Störungen in Vorpommern: Die Studie “Leben und Gesundheit in Vorpommern” (SHIP) und der Bundesgesundheitssurvey 1998 im Vergleich. Gesundheitswesen. (2005) 67:39–47. 10.1055/s-2004-813829 15672305

[54] ATC-Index. *Anatomisch- therapeutisch- chemische Klassifikation mit Tagesdosen: Amtliche Fassung des ATC-Index mit DD-Angaben für Deutschland* (2007).

[55] Swainson MG Batterham AM Tsakirides C Rutherford ZH Hind K. Prediction of whole-body fat percentage and visceral adipose tissue mass from five anthropometric variables. PLoS One. (2017) 12:e0177175. 10.1371/journal.pone.0177175 28493988 PMC5426673

[56] Winter T Friedrich N Lamp S Schäfer C Schattschneider M Bollmann S The integrated research biobank of the university medicine greifswald. Open J Bioresour. (2020) 1–6. 10.5334/ojb.64

[57] Eick C Klinger-König J Zylla S Hannemann A Budde K Henning AK Broad metabolome alterations associated with the intake of oral contraceptives are mediated by cortisol in premenopausal women. Metabolites. (2021) 11:193. 10.3390/metabo11040193 33805221 PMC8064380

[58] Bernstein DP Stein JA Newcomb MD Walker E Pogge D Ahluvalia T Development and validation of a brief screening version of the Childhood Trauma Questionnaire. Child Abuse Negl. (2003) 27:169–90. 10.1016/S0145-2134(02)00541-0 12615092

[59] Häuser W Schmutzer G Brähler E Glaesmer H. Maltreatment in childhood and adolescence: results from a survey of a representative sample of the German population. Dtsch Arztebl Int. (2011) 108:287–94. 10.3238/arztebl.2011.0287 21629512 PMC3103979

[60] Konerding U Kohlmann T Alte D John U. Subjective health complaints, health-related quality of life and physician visits: results of the Study of Health in Pomerania (SHIP). Soz.-Präventivmed. (2006) 51:162–73. 10.1007/s00038-006-0036-x 17191541

[61] Klinger-König J Hertel J Terock J Völzke H Van der Auwera S Grabe HJ. Predicting physical and mental health symptoms: additive and interactive effects of difficulty identifying feelings, neuroticism and extraversion. J Psychosom Res. (2018) 115:14–23. 10.1016/j.jpsychores.2018.10.003 30470312

[62] Kroenke K Spitzer RL. The PHQ-9: a new depression diagnostic and severity measure. Psychiatr Ann. (2002) 32:509–15. 10.3928/0048-5713-20020901-06

[63] American Psychiatric Association. Diagnostic and statistical manual of mental disorders: DSM-IV-TM. Washington: American Psychiatric Association (2005). XXXVII, 886 s.

[64] Beck AT Steer RA Brown G. Manual for the beck depression inventory-II. San Antonio: The Psychological Corporation (1996).

[65] R Core Team. R: A Language and Environment for Statistical Computing. Vienna, Austria (2022). Available from: https://www.R-project.org/

[66] Bulmer MG. Principles of statistics. Newburyport: Dover Publications (2012). 354 p.

[67] Benjamini Y Hochberg Y. Controlling the false discovery rate: a practical and powerful approach to multiple testing. J R Stat Soc B Methodol. (1995) 57:289–300. 10.1111/j.2517-6161.1995.tb02031.x

[68] Revelle W. psych: Procedures for Psychological, Psychometric, and Personality Research. Evanston, Illinois (2022). Available from: https://CRAN.R-project.org/package=psych

[69] Baron RM Kenny DA. The moderator–mediator variable distinction in social psychological research: conceptual, strategic, and statistical considerations. J Pers Soc Psychol. (1986) 51:1173–82. 10.1037/0022-3514.51.6.1173 3806354

[70] Keefe JR Guo W Li QS Amsterdam JD Mao JJ. An exploratory study of salivary cortisol changes during chamomile extract therapy of moderate to severe generalized anxiety disorder. J Psychiatr Res. (2018) 96:189–95. 10.1016/j.jpsychires.2017.10.011 29080520 PMC5710842

[71] Mantella RC Butters MA Amico JA Mazumdar S Rollman BL Begley AE Salivary cortisol is associated with diagnosis and severity of late-life generalized anxiety disorder. Psychoneuroendocrinology. (2008) 33:773–81. 10.1016/j.psyneuen.2008.03.002 18407426 PMC2766671

[72] Norman RE Byambaa M De R Butchart A Scott J Vos T. The long-term health consequences of child physical abuse, emotional abuse, and neglect: a systematic review and meta-analysis. PLoS Med. (2012) 9:e1001349. 10.1371/journal.pmed.1001349 23209385 PMC3507962

[73] Spijker J de Graaf R Bijl RV Beekman AT Ormel J Nolen WA. Duration of major depressive episodes in the general population: results from The Netherlands mental health survey and incidence study (NEMESIS). BJP. (2002) 181:208–13. 10.1192/bjp.181.3.208 12204924

[74] Melartin TK Rytsälä HJ Leskelä US Lestelä-Mielonen PS Sokero TP Isometsä ET. Severity and comorbidity predict episode duration and recurrence of DSM-IV Major depressive disorder. J Clin Psychiatry. (2004) 65:19397. 10.4088/JCP.v65n0612 15291658

[75] Pan A Sun Q Okereke OI Rexrode KM Hu FB. Depression and risk of stroke morbidity and mortality: a meta-analysis and systematic review. JAMA. (2011) 306:1241–9. 10.1001/jama.2011.1282 21934057 PMC3242806

[76] Avila C Holloway AC Hahn MK Morrison KM Restivo M Anglin R An overview of links between obesity and mental health. Curr Obes Rep. (2015) 4:303–10. 10.1007/s13679-015-0164-9 26627487

[77] Carney RM Freedland KE Miller GE Jaffe AS. Depression as a risk factor for cardiac mortality and morbidity. J Psychosom Res. (2002) 53:897–902. 10.1016/S0022-3999(02)00311-2 12377300

[78] Compton MT Daumit GL Druss BG. Cigarette smoking and overweight/obesity among individuals with serious mental illnesses: a preventive perspective. Harv Rev Psychiatry. (2006) 14:212–22. 10.1080/10673220600889256 16912007

[79] Plurphanswat N Kaestner R Rodu B. The effect of smoking on mental health. Am J Health Behav. (2017) 41:471–83. 10.5993/AJHB.41.4.12 28601107

[80] Minichino A Bersani FS Calò WK Spagnoli F Francesconi M Vicinanza R Smoking behaviour and mental health disorders–mutual influences and implications for therapy. Int J Environ Res Public Health. (2013) 10:4790–811. 10.3390/ijerph10104790 24157506 PMC3823321

[81] Boden-Albala B Sacco RL. Lifestyle factors and stroke risk: exercise, alcohol, diet, obesity, smoking, drug use, and stress. Curr Atheroscler Rep. (2000) 2:160–6. 10.1007/s11883-000-0111-3 11122740

[82] Hertel J König J Homuth G Van der Auwera S Wittfeld K Pietzner M Evidence for stress-like alterations in the HPA-axis in women taking oral contraceptives. Sci Rep. (2017) 7:14111. 10.1038/s41598-017-13927-7 29074884 PMC5658328

