# Supplementary material for: Association between childhood maltreatment and adult cortisol concentrations mediated through subjective health complaints
Source: Front Epidemiol. 2023 Feb 17;3:1098822. doi: 10.3389/fepid.2023.1098822 (PMC10911021; doi:10.3389/fepid.2023.1098822)
Supplement: Supplementary file 1 [file Datasheet1.docx]

Supplementary Material

# Figures

**Figure S1.** Selection of the analytic samples.
*CTQ = Childhood Trauma Questionnaire; HbA1c = glycated hemoglobin; WBC = white blood cell*


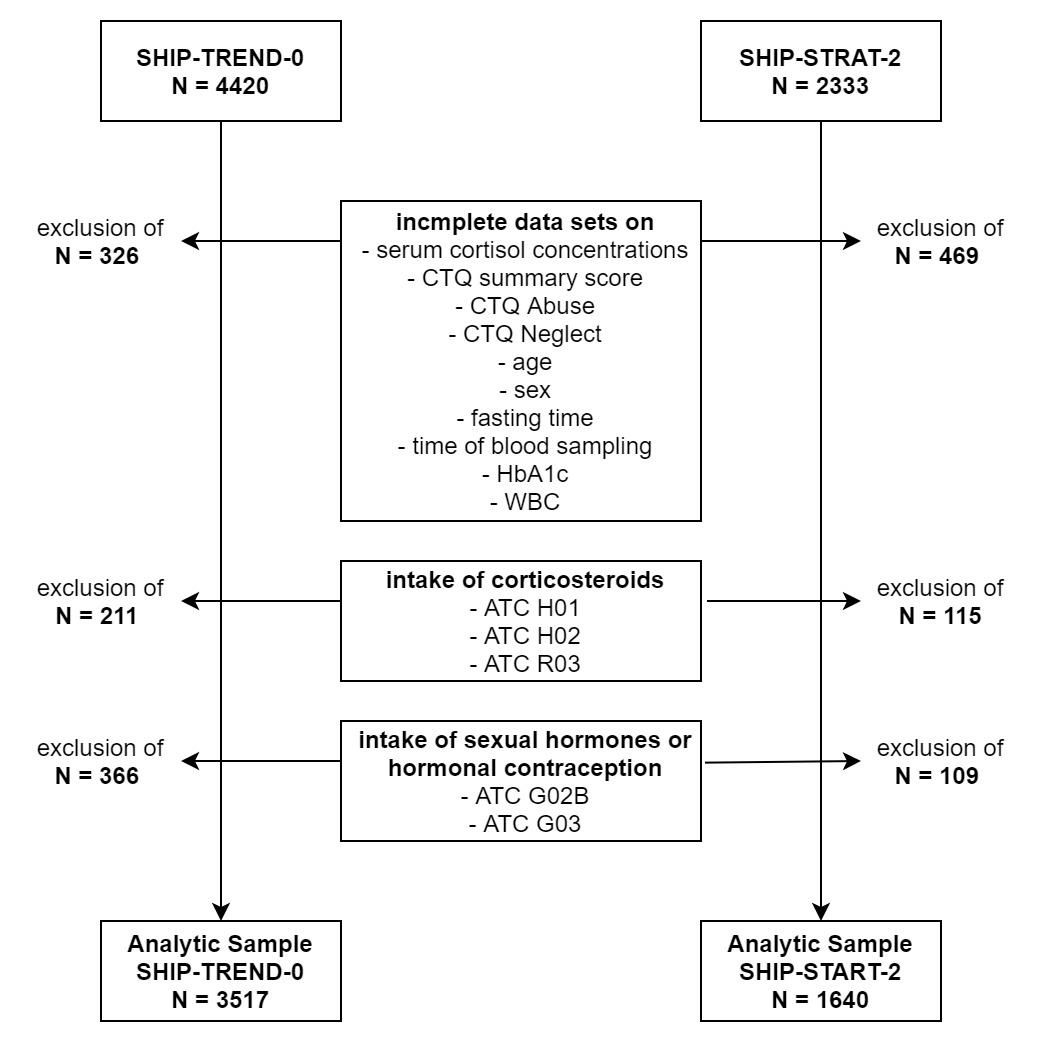


# Tables

**Table S1.** Descriptive statistics of the covariates to compare the initial and the analytic sample of SHIP-TREND-0 and SHIP-START-2.

|  | **Initial Sample** | | **Analytic Symple** | |  |
| --- | --- | --- | --- | --- | --- |
|  | **N** | **M (SD)** | **N** | **M (SD)** | **p-value** |
| **SHIP-TREND-0** |  |  |  |  |  |
| **Age (Years)** | 4420 | 51.96 (15.46) | 3517 | 52.86 (14.76) | 0.012^a^ |
| **Sex (% Female)** | 4420 | 51.47 | 3517 | 46.83 | 4.30e-05^b^ |
| **Fasting time (%)** | 4419 |  | 3517 |  | 0.192^b^ |
| **< 10:00** |  | 42.43 |  | 41.03 |  |
| **10:00 – 12:00** |  | 16.27 |  | 16.38 |  |
| **> 12:00** |  | 41.30 |  | 42.59 |  |
| **Time of Blood Sampling (h:min)** | 4419 | 9:16 (1:06) | 3517 | 9:14 (1:04) | 0.196^a^ |
| **HbA1c (%)** | 4394 | 5.35 (0.75) | 3517 | 5.38 (0.74) | 0.040^a^ |
| **WBC (Gpt/l)** | 4402 | 6.14 (1.77) | 3517 | 6.02 (1.69) | 0.006^a^ |
| **SHIP-START-2** |  |  |  |  |  |
| **Age (Years)** | 2333 | 57.37 (13.66) | 1640 | 58.00 (13.05) | 0.135^a^ |
| **Sex (% Female)** | 2333 | 52.94 | 1640 | 48.90 | 0.013^b^ |
| **Fasting time (%)** | 2330 |  | 1640 |  | 0.442^b^ |
| **< 10:00** |  | 93.73 |  | 94.39 |  |
| **10:00 – 12:00** |  | 1.46 |  | 1.22 |  |
| **> 12:00** |  | 4.81 |  | 4.39 |  |
| **Time of Blood Sampling (h:min)** | 2331 | 9:33 (1:01) | 1640 | 9:33 (1:01) | 0.830^a^ |
| **HbA1c (%)** | 2319 | 5.49 (0.78) | 1640 | 5.51 (0.77) | 0.334^a^ |
| **WBC (Gpt/l)** | 2324 | 6.14 (1.74) | 1640 | 6.02 (1.66) | 0.039^a^ |
| *HbA1c = glycated hemoglobin; WBC = white blood cell count ^a^ Wilcoxon Rank Sum Test; ^b^ Fisher’s Exact Test* | | | | | |

**Table S2.** Associations between the potential mediator variables in SHIP-TREND-0 and SHIP-START-2.

|  | **Hypertension** | **WHtR** | **Obesity** | **Triglycerides** | **HDL-C** | **Depr Symp** | **Mental SHC** | **Somatic SHC** | **Alcohol** | **Risky Alcohol** | **Packyears** | **Ever Smoker** |
| --- | --- | --- | --- | --- | --- | --- | --- | --- | --- | --- | --- | --- |
| **SHIP-TREND-0** | | | | | | | | | | | | |
| **Systolic BP** | 0.04 | 0.032 | 0.05 | 0.033 | -0.017 | -0.007 | -0.028 | -0.030 | 0.007 | -1.49e-02 | -0.025 | 0.03 |
| **Hypertension** |  | 1.00 *** | 267.19 *** | 0.49 *** | -2.63e-01 *** | 0.01 | 0.19 *** | 0.54 *** | -1.04e-01 ** | 2.57 | 0.50 *** | 15.36 *** |
| **WHtR** |  |  | 2.16 *** | 0.346 *** | -0.328 *** | 0.015 | 0.052 | 0.217 *** | -0.016 | 0.07 | 0.290 *** | 0.04 |
| **Obesity** |  |  |  | 0.64 *** | -5.37e-01 *** | 0.10 ** | 0.13 *** | 0.44 *** | -1.12e-01 ** | 1.34 | 0.32 *** | 0.75 |
| **Triglycerides** |  |  |  |  | -0.472 *** | 0.009 | 0.024 | 0.067 ** | 0.022 | 0.18 ** | 0.170 *** | 0.24 *** |
| **HDL-C** |  |  |  |  |  | 0.028 | 0.015 | -0.045 | 0.077 *** | 0.19 ** | -0.148 *** | -3.00e-01 *** |
| **Depr Symp** |  |  |  |  |  |  | 0.724 *** | 0.463 *** | -0.026 | 0.09 | 0.005 | 0.12 *** |
| **Mental SHC** |  |  |  |  |  |  |  | 0.664 *** | -0.052 | -6.91e-02 | 0.034 | 0.07 |
| **Somatic SHC** |  |  |  |  |  |  |  |  | -0.074 *** | -8.74e-02 | 0.097 ** | 0.02 |
| **Alcohol** |  |  |  |  |  |  |  |  |  | 4.46 *** | 0.167 *** | 0.40 *** |
| **Risky Alcohol** |  |  |  |  |  |  |  |  |  |  | 0.40 *** | 58.80 *** |
| **SHIP-START-2** | | | | | | | | | | | | |
| **Systolic BP** | 0.07 | 0.047 | 0.10 * | 0.121 *** | -0.042 | -0.032 | -0.003 | -0.021 | 0.022 | -3.96e-02 | -0.001 | -3.13e-04 |
| **Hypertension** |  | 0.90 *** | 131.50 *** | 0.40 *** | -3.74e-01 *** | 0.18 *** | 0.27 *** | 0.44 *** | -1.10e-01 * | 1.32 | -3.39e-02 | 5.29 * |
| **WHtR** |  |  | 2.07 *** | 0.309 *** | -0.384 *** | 0.007 | -0.031 | 0.170 *** | -0.004 | 0.08 | -0.032 | 0.12 * |
| **Obesity** |  |  |  | 0.55 *** | -5.58e-01 *** | 0.01 | -5.68e-02 | 0.28 *** | -6.99e-02 | 0.05 | -7.36e-02 | 3.36 |
| **Triglycerides** |  |  |  |  | -0.501 *** | -0.052 | -0.046 | 0.038 | 0.080 | 0.30 *** | -0.012 | 0.13 * |
| **HDL-C** |  |  |  |  |  | 0.041 | 0.068 | -0.052 | -0.001 | -4.41e-02 | -0.039 | -2.36e-01 *** |
| **Depr Symp** |  |  |  |  |  |  | 0.613 *** | 0.446 *** | -0.062 | -6.52e-02 | -0.020 | 0.02 |
| **Mental SHC** |  |  |  |  |  |  |  | 0.709 *** | 0.002 | 0.01 | 0.035 | -5.58e-02 |
| **Somatic SHC** |  |  |  |  |  |  |  |  | -0.045 | -3.57e-03 | -0.015 | 0.03 |
| **Alcohol** |  |  |  |  |  |  |  |  |  | 4.47 *** | 0.005 | 0.30 *** |
| **Risky Alcohol** |  |  |  |  |  |  |  |  |  |  | 0.05 | 18.54 *** |
| *BP = blood pressure; WHtR = waist-height ratio; HDL-C = high-density lipoprotein cholesterol; Depr Symp. = Depressive Symptoms; SHC = Subjective Health Complaints; Alcohol = Alcohol Consumption; Risky Alcohol = Risky Alcohol Consumption Pearson’s r is reported for two continuous variables. Cohen’s d is reported for one continuous and one dichotomous variable. χ² is reported for two dichotomous variables. * p < .05; ** p < .01; *** p < .001* | | | | | | | | | | | | |

**Table S3.** Sensitivity Analyses: Separate mediation analyses for the impact of the CTQ summary score, abuse and neglect on serum cortisol concentrations by the symptoms of the subjective health complaints questionnaire.

|  | **Total Effect** | | | **Direct Effect** | | | **Indirect Effect** | | |  |
| --- | --- | --- | --- | --- | --- | --- | --- | --- | --- | --- |
|  | **β** | **SE** | **p-value** | **β** | **SE** | **p_FDR_** | **β** | **SE** | **p_FDR_** | **Proportion Mediated** |
| **CTQ Summary Score** | -0.052 | 0.013 | 5.70e-05 |  |  |  |  |  |  |  |
| *Exhaustion* |  |  |  |  |  |  |  |  |  |  |
| **Faintness** |  |  |  | -0.046 | 0.013 | 6.40e-04 | -0.007 | 0.002 | 0.010 | 12.93 |
| **Excessive need for Sleep** |  |  |  | -0.045 | 0.013 | 6.82e-04 | -0.007 | 0.002 | 0.010 | 13.72 |
| **Tiredness** |  |  |  | -0.045 | 0.013 | 6.60e-04 | -0.007 | 0.002 | 0.010 | 13.56 |
| *Difficulty Breathing* |  |  |  |  |  |  |  |  |  |  |
| **Shortness of Breath** |  |  |  | -0.048 | 0.013 | 3.84e-04 | -0.005 | 0.001 | 0.010 | 8.70 |
| *Pain* |  |  |  |  |  |  |  |  |  |  |
| **Back Pain** |  |  |  | -0.049 | 0.013 | 3.84e-04 | -0.004 | 0.002 | 0.028 | 7.32 |
| **Neck/Shoulder Pain** |  |  |  | -0.049 | 0.013 | 3.84e-04 | -0.004 | 0.001 | 0.010 | 6.77 |
| **Headache/Facial Pain** |  |  |  | -0.049 | 0.013 | 3.84e-04 | -0.003 | 0.002 | 0.044 | 6.41 |
| **Joint Pain /Pain in the Limbs** |  |  |  | -0.049 | 0.013 | 3.84e-04 | -0.003 | 0.001 | 0.035 | 5.86 |
| *Disturbances of Sensations* |  |  |  |  |  |  |  |  |  |  |
| **Heaviness/Tiredness in the Legs** |  |  |  | -0.047 | 0.013 | 4.19e-04 | -0.005 | 0.002 | 0.020 | 9.65 |
| **Restless Legs** |  |  |  | -0.048 | 0.013 | 3.84e-04 | -0.004 | 0.002 | 0.026 | 8.49 |
| **Numbness** |  |  |  | -0.049 | 0.013 | 3.84e-04 | -0.003 | 0.002 | 0.045 | 5.96 |
| **CTQ Abuse** | -0.038 | 0.013 | 0.004 |  |  |  |  |  |  |  |
| *Exhaustion* |  |  |  |  |  |  |  |  |  |  |
| **Faintness** |  |  |  | -0.032 | 0.013 | 0.015 | -0.006 | 0.002 | 0.012 | 14.69 |
| **Excessive need for Sleep** |  |  |  | -0.032 | 0.013 | 0.015 | -0.005 | 0.002 | 0.012 | 13.73 |
| **Tiredness** |  |  |  | -0.033 | 0.013 | 0.015 | -0.005 | 0.002 | 0.012 | 13.49 |
| *Difficulty Breathing* |  |  |  |  |  |  |  |  |  |  |
| **Shortness of Breath** |  |  |  | -0.034 | 0.013 | 0.013 | -0.003 | 0.001 | 0.028 | 8.65 |
| *Pain* |  |  |  |  |  |  |  |  |  |  |
| **Back Pain** |  |  |  | -0.034 | 0.013 | 0.013 | -0.003 | 0.001 | 0.036 | 8.69 |
| **Neck/Shoulder Pain** |  |  |  | -0.035 | 0.013 | 0.013 | -0.002 | 0.001 | 0.036 | 6.38 |
| **Headache/Facial Pain** |  |  |  | -0.035 | 0.013 | 0.013 | -0.002 | 0.001 | 0.074 | 5.95 |
| **Joint Pain /Pain in the Limbs** |  |  |  | -0.036 | 0.013 | 0.013 | -0.001 | 8.83e-04 | 0.108 | 3.78 |
| *Disturbances of Sensations* |  |  |  |  |  |  |  |  |  |  |
| **Heaviness/Tiredness in the Legs** |  |  |  | -0.034 | 0.013 | 0.013 | -0.003 | 0.001 | 0.036 | 7.91 |
| **Restless Legs** |  |  |  | -0.035 | 0.013 | 0.013 | -0.002 | 0.001 | 0.039 | 6.56 |
| **Numbness** |  |  |  | -0.035 | 0.013 | 0.013 | -0.002 | 0.001 | 0.087 | 4.89 |
| *Models were adjusted for age (non-linear), sex, fasting time (non-linear), time of blood sampling (non-linear), glycated hemoglobin and white blood cell count. FDR = false discovery rate to adjust for multiple testing; CTQ = Childhood Trauma Questionnaire* | | | | | | | | | | |

**Table S3 [continued].** Sensitivity Analyses: Separate mediation analyses for the impact of the CTQ summary score, abuse and neglect on serum cortisol concentrations by the symptoms of the subjective health complaints questionnaire.

|  | **Total Effect** | | | **Direct Effect** | | | **Indirect Effect** | | |  |
| --- | --- | --- | --- | --- | --- | --- | --- | --- | --- | --- |
|  | **β** | **SE** | **p-value** | **β** | **SE** | **p_FDR_** | **β** | **SE** | **p_FDR_** | **Proportion Mediated** |
| **CTQ Neglect** | -0.037 | 0.013 | 0.005 |  |  |  |  |  |  |  |
| *Exhaustion* |  |  |  |  |  |  |  |  |  |  |
| **Faintness** |  |  |  | -0.032 | 0.013 | 0.014 | -0.004 | 0.001 | 0.038 | 11.01 |
| **Excessive need for Sleep** |  |  |  | -0.031 | 0.013 | 0.017 | -0.004 | 0.001 | 0.038 | 10.25 |
| **Tiredness** |  |  |  | -0.032 | 0.013 | 0.014 | -0.003 | 0.001 | 0.038 | 8.65 |
| *Difficulty Breathing* |  |  |  |  |  |  |  |  |  |  |
| **Shortness of Breath** |  |  |  | -0.034 | 0.013 | 0.014 | -0.002 | 0.001 | 0.049 | 6.53 |
| *Pain* |  |  |  |  |  |  |  |  |  |  |
| **Back Pain** |  |  |  | -0.034 | 0.013 | 0.014 | -0.003 | 0.001 | 0.046 | 7.77 |
| **Neck/Shoulder Pain** |  |  |  | -0.035 | 0.013 | 0.014 | -0.002 | 9.52e-04 | 0.103 | 4.49 |
| **Headache/Facial Pain** |  |  |  | -0.035 | 0.013 | 0.014 | -0.002 | 0.001 | 0.160 | 4.46 |
| **Joint Pain /Pain in the Limbs** |  |  |  | -0.035 | 0.013 | 0.014 | -0.002 | 9.79e-04 | 0.125 | 4.24 |
| *Disturbances of Sensations* |  |  |  |  |  |  |  |  |  |  |
| **Heaviness/Tiredness in the Legs** |  |  |  | -0.033 | 0.013 | 0.014 | -0.003 | 0.001 | 0.053 | 6.98 |
| **Restless Legs** |  |  |  | -0.034 | 0.013 | 0.014 | -0.003 | 0.001 | 0.046 | 7.40 |
| **Numbness** |  |  |  | -0.034 | 0.013 | 0.014 | -0.002 | 1.00e-05 | 0.103 | 4.76 |
| *Models were adjusted for age (non-linear), sex, fasting time (non-linear), time of blood sampling (non-linear), glycated hemoglobin and white blood cell count. FDR = false discovery rate to adjust for multiple testing; CTQ = Childhood Trauma Questionnaire* | | | | | | | | | | |
